# Detection and management of depression in adult primary care patients in Hong Kong: a cross-sectional survey conducted by a primary care practice-based research network

**DOI:** 10.1186/1471-2296-15-30

**Published:** 2014-02-12

**Authors:** Weng Yee Chin, Kit TY Chan, Cindy LK Lam, Samuel YS Wong, Daniel YT Fong, Yvonne YC Lo, Tai Pong Lam, Billy CF Chiu

**Affiliations:** 1 Department of Family Medicine and Primary Care, The University of Hong Kong, Hong Kong, China; 2 Division of Family Medicine and Primary Health Care, School of Public Health and Primary Care, The Chinese University of Hong Kong, Hong Kong, China; 3 School of Nursing, The University of Hong Kong, Hong Kong, China; 4 Department of Family Medicine, Hong Kong West Cluster, Hospital Authority, Hong Kong, China; 5 Family Medicine and Primary Care Centre, Hong Kong Sanatorium and Hospital, Hong Kong, China

**Keywords:** Mental health, Chinese, Depression, Epidemiology, Primary care, Prevalence, Screening, Detection, Practice-based research network

## Abstract

**Background:**

This study aimed to examine the prevalence, risk factors, detection rates and management of primary care depression in Hong Kong.

**Methods:**

A cross-sectional survey containing the PHQ-9 instrument was conducted on waiting room patients of 59 primary care doctors. Doctors blinded to the PHQ-9 scores reported whether they thought their patients had depression and their management.

**Results:**

10,179 patients completed the survey (response rate 81%). The prevalence of PHQ-9 positive screening was 10.7% (95% CI: 9.7%-11.7%). Using multivariate analysis, risk factors for being PHQ-9 positive included: being female; aged ≤34 years; being unmarried; unemployed, a student or a homemaker; having a monthly household income < HKD$30,000 (USD$3,800); being a current smoker; having no regular exercise; consulted a doctor or Chinese medical practitioner within the last month; having ≥ two co-morbidities; having a family history of mental illness; and having a past history of depression or other mental illness. Overall, 23.1% of patients who screened PHQ-9 positive received a diagnosis of depression by the doctor. Predictors for receiving a diagnosis of depression included: having higher PHQ-9 scores; a past history of depression or other mental health problem; being female; aged ≥35 years; being retired or a homemaker; being non-Chinese; having no regular exercise; consulted a doctor within the last month; having a family history of mental health problems; and consulted a doctor in private practice.

In patients diagnosed with depression, 43% were prescribed antidepressants, 11% were prescribed benzodiazepines, 42% were provided with counseling and 9% were referred, most commonly to a counselor.

**Conclusion:**

About one in ten primary care patients screen positive for depression, of which doctors diagnose depression in approximately one in four. At greatest risk for depression are patients with a past history of depression, who are unemployed, or who have multiple illnesses. Patients most likely to receive a diagnosis of depression by a doctor are those with a past history of depression or who have severe symptoms of depression. Chinese patients are half as likely to be diagnosed with depression as non-Chinese patients. Over half of all patients diagnosed with depression are treated with medications.

## Background

Depression is a common and serious disorder that impairs quality of life [1]. There are many barriers to recognizing and managing patients with depression, particularly in primary care where most patients with depressive disorders are seen [2-4]. Around the world, the prevalence of depressive disorders in primary care has been estimated to be between 10-20%, of which around half remain undetected by doctors [5]. A recent household telephone survey conducted on a Hong Kong Chinese general population sample estimated the cross-sectional community prevalence for moderate to severe depression (defined as PHQ-9 score >9) as 4.3%, however only 1.6% reported to have had a lifetime history of depression diagnosed by a doctor [6]. Similarly, in the 2003–2004 Hong Kong Population Health Survey, only 1.5% of adult respondents reported having a past diagnosis of depression by a doctor [7].

Primary care is the entry point for most people into the healthcare system and a common pathway to mental health care services. The choice of interventions offered by primary care physicians (PCPs) can have a significant impact both on patients’ quality of life as well as on health service demands. Not all primary care patients with depression respond to antidepressants and current international guidelines on adult primary care depression recommend a ‘stepped’ approach which may involve watchful waiting, counselling, use of psychotropic medications, or referral for further psychological or psychiatric services. There is evidence that delivery of stepped care can improve patient outcomes [8-10].

Hong Kong is a Special Administrative Region of the People's Republic of China located in the Pearl Delta district, south of the Guangdong province of China. It has a population of approximately seven million of whom 95% are of Chinese descent. As a result of its past as a British colony, Hong Kong is often referred to as a place where “East meets West”. Despite this, the Chinese living in Hong Kong still hold many of the values, beliefs and behaviours typical of Chinese populations both from China and overseas which have their origins based on Taoism and Confucianism [11]. Hong Kong has a developed capitalist economy, with a gross domestic product of US$301.6 billion of which about 5.5% is spent on healthcare and about 0.24% on mental health [12]. It has a two-tiered mixed medical economy and a pluralistic healthcare system. 80% of primary care is delivered through the ‘fee-for-service’ private sector with the remaining 20% delivered through government-funded General Out-Patient Clinics (GOPCs) which cater mainly for chronic disease management and for the elderly. In the private ‘fee-for-service’ setting, any patient can seek the care of any doctor for any illness, and there are no statutory post-graduate qualifications required for doctors to practice in primary care [13]. As little is currently known about the epidemiology, detection and management patterns for depression in Hong Kong’s primary care setting, the objectives of this study are to:

1. Estimate the cross-sectional prevalence of depression in primary care and its associated risk factors;

2. Examine the detection rates for depression and the predictors for diagnosis of depression by a doctor;

3. Examine how primary care doctors manage patients with depression.

The findings of this study will be used to help guide mental health service planning and policy development, and will contribute to the overall knowledge on identification and management of depression in Chinese primary care patients.

## Methods

### Study design

This was a cross-sectional observational study using a primary care practice-based research network. This study formed the baseline for a longitudinal cohort study examining the outcomes of depression in primary care [14].

### Participants and sampling

Primary care physicians (PCPs) working in clinics across Hong Kong were invited to join our research network. Doctors were recruited using the mailing list of the Hong Kong College of Family Physicians (HKCFP) and consisted of PCPs working in solo and group private practice, government-funded General Out-Patient Clinics of the Hospital Authority of Hong Kong, and non-profit, non-governmental organizations (NGO).

All eligible patients presenting to the study doctor on one randomly selected day each month over a 12-month recruitment period were invited to participate. The recruitment days were randomly generated but excluded Sundays and public holidays. A twelve-month baseline collection period was used to account for seasonal variability and to improve representation of the primary care case load. All consecutive waiting room patients were approached to join the study. Patients were excluded if they were <18 years, did not speak or understand English, Cantonese or Mandarin, had cognitive or communication difficulties, had already been recruited to the study, or did not consult the study doctor.

A coded, anonymous questionnaire containing items on age, gender, marital status, ethnicity, education, household income, district of residence, co-existing medical conditions, family history of mental illness, previous doctor-diagnosed depression or other mental illness, recent mental health or other health care service use, and the PHQ-9 was self-completed by participants in the PCP’s waiting room. Questionnaires were available in English and Chinese. A bilingual research assistant was present to explain the study, obtain consent, and distribute and collect questionnaires. If subjects had difficulty completing the questionnaire, the research assistant helped to administer the questionnaire. Doctors who were blinded to the questionnaire results were asked to complete a clinical data collection form for each patient recruited into the study.

### Study instruments

A. The Patient Health Questionnaire 9 (PHQ-9). The PHQ-9 is a self-reported depression component of the Primary Care Evaluation of Mental Disorder Procedure (PRIME-MD) [15] which has been validated for use in primary care for diagnosis of depression. It scores each of the 9 DSM-IV criteria from 0 (not at all) to 3 (nearly every day). It has been calibrated against the Chinese Hamilton Depression Scale for screening use in Hong Kong’s primary care setting. Using a cut-off score of 9, the PHQ-9 has a sensitivity of 80% and specificity of 92% for the diagnosis of depression [16]. The PHQ-9 can also be used to evaluate the severity of symptoms (score 1–4 minimal, 5–9 mild, 10–14 moderate, 15–19 moderately severe, 20–27 severe) and has been used for monitoring symptom progression or remission over time [17].

B. Personal data questions on socio-demography and co-morbidity were adapted from previous primary care patient surveys performed in Hong Kong [18].

C. Doctor’s data collection form. Doctors were asked to record the patient’s presenting problem, their opinion on whether they felt the patient had depression operationally defined as a “clinically significant depressive disorder”, and whether the diagnosis was new or old. If the doctor reported a diagnosis of depression, the doctor was asked to provide further information regarding the duration of illness, and their management.

### Sample size calculation

Details of the sample size estimation required to calculate the outcome measures of the cross-sectional and longitudinal cohort study have already been described in our published study protocol [14]. For the estimation of the prevalence of depression by PHQ-9, the sample size was estimated to ensure an error of <2% for an anticipated prevalence of 20%. Without the consideration of the design effect due to cluster sampling by practice, 1540 subjects were needed in total with a 95% confidence interval. The intraclass correlation (ICC) for the intra-cluster correlation was taken as 0.02 which was more conservative than the one reported in the Diamond study [19]. With 50 practices and after accounting the design effect due to cluster sampling, 80 subjects per practice were needed.

### Analysis

The prevalence of depression was estimated from the patient’s screening PHQ-9 scores (using a cut-off score >9 to define a positive case) with a 95% confidence interval taking into account the clustering by practice effect [12]. Chi-square and multiple logistic regression analyses were conducted to identify the patient risk factors associated with PHQ-9 positive screening and the patient predictors for being diagnosed with depression by the doctor. As demographic risk factor identification was the main goal of analysis, all demographic factors were kept in the regression model, whilst other patient clinical characteristics (such as PHQ-9 severity, patient-reported past history of depression and patient-reported past history of other mental illness) were entered in the regression model as a second block to allow for separate comparison. These patient characteristics were examined in separate blocks as they were considered to be theoretically highly linked to the dependent variables, and their inclusion together may result in a reduction of the significance of the other demographic factors which we wished to assess. Multicollinearity diagnostics were performed for all regression models [20]. Only patients with full data were used in the model. Imputation or other substitution methods were not used as there was a sufficiently large sample size to perform all analyses. Management of depression was descriptively analyzed based on the doctor’s reported management of patients who they diagnosed with depression. All analyses were performed using SPSS 21.

### Ethics approvals

This study received approvals by the Institutional Review Board of the University of Hong Kong/Hospital Authority Hong Kong West Cluster, the Research Committee of Evangel Hospital, the Research Committee of Hong Kong Sanatorium and Hospital, the Research Committee of Matilda International Hospital, the Research Ethics Committee for Hong Kong Hospital Authority Kowloon East Cluster and Kowloon Central Cluster, and the Joint Chinese University of Hong Kong and Hong Kong Hospital Authority New Territories East Cluster Clinical Ethics Review Committee.

## Results

### Study subjects

Fifty-nine PCPs participated in this study. The doctors’ clinics were located across all three geographical regions of Hong Kong (Hong Kong Island, Kowloon and New Territories). Forty four of the study doctors had Fellowship qualifications in General Practice (or equivalent); one had a Fellowship qualification in Surgery; one had a Fellowship qualification in Emergency Medicine; whilst the remaining doctors had no postgraduate specialty qualifications. Fourteen of the study doctors had completed a Post-Graduate Diploma in Psychological Medicine.

Recruitment of patients occurred between October 2010 and January 2012. A total of 10,179 patients consented to participate with a response rate of 81% of all eligible subjects approached. Public sector Government Out-patient Clinic (GOPC) patients made up 26% of the subjects, whilst the remainder were recruited from private practices and Non-Governmental Organizations (NGOs) aligning proportionately with the overall delivery of primary care in Hong Kong [21]. Amongst all subjects, the mean age was 49 years; 95.7% were ethnically Chinese; and 56.6% were female. Demographic information on the doctors, clinics and subjects are shown in Table 1.

**Table 1:** Demographic information on doctors and patient

**Doctors and clinic: (N = 59)**	**Patient demographic: (N = 10.179)**
**Gender**	**Gender**
Male 42 (712%)	Male 40.7%
Female 17 (28.8%)	Female 56.6%
**Age**	**Age**
Mean age 44.9 years (range 30–75 years)	Mean age 49.0 years (range 18–103 years)
	18-24 years 6.8%
**Place of graduation**	25-24 years 18.4%
Hong Kong 46 (78.0%)	35-44 years 16.9%
Overseas 13 (22.0%)	45-54 years 17.5%
(China 2; UK 3; Australia/NZ 7; other 1)	55-64 years15.7%
	65+ years 20.3%
	**Ethnicity**
**Post-graduate qualifications**	Chinese 95.7%
Fellowship in family medicine (or equivalent) 44 (74.6%)	Non-Chinese 3.9%
	**Household monthly income (HKD)**
Diploma in psychological medicine 14 (23.7%)	≤$5000 12.2%
Location of clinic*	$5,001-$10,000 7.5%
Hong Kong Island 26 (44.1%)	$10,001-$20,000 17.8%
Kowloon 18 (30.5%)	$20,001-$30,000 15.1%
New Territories 15 (25.4%)	$30,001-$40,000 10.5%
	>$40,000 21.2%
	**Marital status**
	Single 26.6%
**Type of practice**	Married59.8%
Private solo 21 (35.6%)	Widowed 7.6%
Private group 12 (20.3%)	Separated/divorced 3.2%
Private hospital 11 (18.6%)	**Education level**
Government clinic 11 (18.6%)	No formal schooling 7.7%
NGO 3 (5.1%)	Primary 16.0%
University 1^†^ (1.7%)	Secondary 40.5%
	Tertiary 32.7%
	District of Residence*
	Hong Kong Island 40.1%
	Kowloon 22.0%
	New Territories 34.1%
	Mainland China 0.3%

*Hong Kong is divided into three major geographical regions: Hong Kong Island, Kowloon and the New Territories.

^†^One study doctor changed practice type from private solo to university health services during the study period.

### Prevalence of depressive symptoms

Using a PHQ-9 cut-off score > 9 to define a positive case and taking into account the effect of clustering by doctor, the prevalence for PHQ-9 positive screened depression was estimated at 10.7% (95% CI: 9.7%-11.7%). The prevalence was higher in patients recruited from public sector settings (12.0%) than private (10.1%); in women (12.2%) than men (8.1%); and in patients under the age of 35 years (12.3%) than those who were 35 years or over (9.8%). Mean PHQ-9 scores were higher in women than men in all age groups. Mean PHQ-9 scores became lower with increasing age in both sexes in a step-wise fashion as shown in Table 2.

**Table 2:** PHQ-9 severity and mean scores among study participants (N = 10,179)

	**Overall**	**Male**	**Female**
**PHQ-9 scores by severity (score range)**			
Minimal (0–4)	61.1%	65.4%	59.0%
Mild (5–9)	25.3%	24.5%	26.0%
Moderate (10–14)	7.5%	6.0%	8.3%
Moderately severe (15–19)	2.3%	1.6%	2.8%
Severe (20–27)	0.8%	0.6%	1.0%
**Mean PHQ-9 scores by age and sex**			5.42
18-24 yrs	5.33	5.20	5.23
25-34 yrs	5.09	4.87	4.61
35-44 yrs	4.44	4.20	4.55
45-54 yrs	4.09	3.49	4.09
55-64 yrs	3.68	3.14	4.09
65+ yrs	3.57	3.01	3.98

*Note.* PHQ-9 scale score ranges from 0 to 27 with higher score indicating more frequent occurrence of depressive symptoms. A person scoring 10 or above is classified as ‘PHQ-9screened positive’.

Total count can be less than 100% due to missing information provided.

### Patient factors associated with PHQ-9 positive screening

Patient factors associated with PHQ-9 positive screening, the corresponding chi-square statistics, adjusted odds ratios (OR) derived from multiple logistic regression analyses, and model evaluation statistics are shown in Table 3. Risk factors for PHQ-9 positive screening included being female; being aged 18–34 (relative to aged 55 above); being unmarried; being unemployed, a student or a homemaker (relative to being employed); having a household income under HKD$30,000 per month; being a current smoker; having no exercise habit; having consulted a doctor or Chinese medical practitioner in the preceding four weeks; having ≥ two co-morbidities; and having a family history of mental health problems. At highest risk for being PHQ-9 positive were patients who were unemployed (2.74-fold greater increased risk than employed patients), and patients with ≥ two co-morbidities (2.45-fold increased risk than patients with no co-existing illness). All patient demographic factors which were significant in the chi-square analysis remained statistically significant in the multiple logistic regression analysis except for service sector.

**Table 3:** Associations between patient factors and PHQ-9 screening outcome

**Patient factor**	**PHQ + ve, n (%)**	**PHQ-ve, n (%)**	**χ2 (df)**	**Multiple logistic regression (*n*** **= 7675)**^**a**^	
				**Wald χ2 (df)**^**b**^**/Adj. OR (95% CI)**	
**Gender**			44.41 (1)**	11.24 (1)**	4.55 (1)*
Male	336 (32.4)	3721 (43.2)		1.00	1.00
Female	702 (67.6)	4897 (56.8)		1.36 (1.14 - 1.62)	1.22 (1.02 - 1.46)
**Age group**			17.11 (2)**	9.99 (2)**	11.89 (2)**
18-34 yrs	316 (31.1)	2201 (26.0)		1.00	1.00
35-54 yrs	371 (36.5)	3045 (35.9)		0.95 (0.76 - 1.18)	0.87 (0.70 - 1.09)
55+ yrs	329 (32.4)	3232 (38.1)		0.63 (0.46 - 0.87)	0.58 (0.42 - 0.80)
**Education**			0.45 (1)	*0.01 (1)*	*0.09 (1)*
Secondary or above	772 (75.0)	6514 (75.9)		1.00	1.00
Primary or no formal education	258 (25.0)	2069 (24.1)		0.99 (0.78 - 1.25)	1.04 (0.82 - 1.32)
**Marital status**			71.62 (1)**	28.18 (1)**	24.14 (1)**
Married	508 (49.3)	5394 (62.9)		1.00	1.00
Single/separated/divorced/widowed	523 (50.7)	3188 (37.1)		1.62 (1.35 - 1.93)	1.57 (1.31 - 1.88)
**Working Status**			83.98 (4)**	32.09 (4)**	22.70 (4)**
Employed	582 (57.6)	5395 (63.5)		1.00	1.00
Unemployed	48 (4.7)	116 (1.4)		2.74 (1.79 - 4.18)	2.31 (1.48 - 3.60)
Retired	201 (19.8)	1833 (21.6)		0.97 (0.72 - 1.31)	0.95 (0.70 - 1.29)
House-maker	129 (12.7)	899 (10.6)		1.33 (1.01 - 1.75)	1.30 (0.98 - 1.73)
Student	52 (5.1)	248 (2.9)		1.70 (1.17 - 2.47)	1.62 (1.11 - 2.37)
**Household monthly income**			75.00 (1)**	28.93 (1)**	27.23 (1)**
More than HK$30,000	220 (24.6)	2951 (39.4)		1.00	1.00
HK30,000 or below	676 (75.4)	4539 (60.6)		1.66 (1.38 - 2.00)	1.65 (1.37 - 2.00)
**Ethnicity**			0.01 (1)	*1.08 (1)*	*0.16 (1)*
Chinese	1039 (96.3)	8471 (96.3)		1.00	1.00
Non-Chinese	40 (3.7)	321 (3.7)		1.27 (0.81 - 1.98)	1.10 (0.69 - 1.74)
**Smoking habit**			19.24 (1)**	10.09 (1)**	8.90 (1)**
Non- or ex-smoker	847 (82.6)	7511 (87.4)		1.00	1.00
Current smoker	179 (17.4)	1079 (12.6)		1.44 (1.15 - 1.81)	1.42 (1.13 - 1.79)
**Alcohol use**			0.18 (1)	*0.40 (1)*	*0.26 (1)*
Less than once a week or never	904 (87.9)	7510 (87.4)		1.00	1.00
More than once a week	125 (12.1)	1084 (12.6)		1.08 (0.85 - 1.39)	1.07 (0.83 - 1.38)
**Regular exercise pattern**			57.12 (1)**	29.46 (1)**	
Yes	647 (63.2)	6361 (74.3)		1.00	1.00
No exercise at all	377 (36.8)	2204 (25.7)		1.58 (1.34 - 1.86)	1.58 (1.33 - 1.86)
**Seen a doctor in past 4 weeks**			94.67 (1)**	41.12 (1)**	30.88 (1)**
No	356 (33.0)	4276 (48.7)		1.00	1.00
More than once	723 (67.0)	4512 (51.3)		1.70 (1.45 - 2.00)	1.60 (1.36 - 1.89)
**Seen a TCM practitioner in past 4 weeks**			87.93 (1)**	32.20 (1)**	31.28 (1)**
No	751 (69.6)	7176 (81.6)		1.00	1.00
More than once	328 (30.4)	1615 (18.4)		1.70 (1.41 - 2.03)	1.70 (1.41 - 2.05)
**Disease co-morbidity**			71.11 (2)**	63.57 (2)**	48.96 (2)**
None	437 (42.3)	4319 (50.2)		1.00	1.00
One	223 (21.6)	2207 (25.7)		1.25 (1.01 - 1.55)	1.19 (0.96 - 1.48)
Two or more	374 (36.2)	2071 (24.1)		2.45 (1.95 - 3.08)	2.22 (1.76 - 2.80)
**Family history mental illness**			37.88 (1)**	31.05 (1)**	16.62 (1)**
No	869 (85.8)	7716 (91.6)		1.00	1.00
Yes	144 (14.2)	704 (8.4)		1.89 (1.51 - 2.36)	1.62 (1.28 - 2.04)
**Service sector**			8.37 (1)**	*2.49 (1)*	
Public	318 (29.5)	2232 (25.4)		1.00	1.00
Private	761 (70.5)	6560 (74.6)		0.85 (0.70 - 1.04)	0.85 (0.70 - 1.05)
**Self-reported depression history**^**c**^			420.70 (1)**		125.97 (1)**
No	795 (78.0)	8104 (95.1)			1.00
Yes	224 (22.0)	420 (4.9)			4.10 (3.20 - 5.24)
**Self-reported other mental illness history**^**c**^			110.70 (1)**		4.82 (1)*
No	888 (91.1)	8195 (97.4)			1.00
Yes	87 (8.9)	219 (2.6)			1.51 (1.05 - 2.17)

*p < .05; **p < .01.

^a^Model Chi-square = 546.73, df = 22, *p* < .001; Nagelkerke R-square = 0.143; Hosmer & Lemeshow Test, Chi-square = 14.42, df = 8, *p* = 0.072.

^b^Wald test statistics are shown in italic.

^c^Factors entered in second block of the regression model; Block Chi-square = 141.46, df = 2, p < .001.

With all patient demographic variables retained in block 1, two more patient characteristics were then included in the second block to assess for additional significance: ‘patient-reported previous diagnosis of depression’ and ‘patient-reported previous diagnosis of other mental illnesses’. The presence of a previous diagnosis of depression was associated with a 4.1-fold increased risk of being PHQ-9 positive, whilst the presence of a previous diagnosis of other mental illness was associated with a 1.5-fold increased risk. All patient demographic factors that were significant in the previous analysis remained significant with the inclusion of block 2 variables. Multicollinearity was not evident among the factors analyzed as demonstrated by all variance inflation factors being <4 and no condition indices >30. The predictors as a set explained 14.3% of model variance (Nagelkerke R-square = 0.143); the model chi-square testing was significant and Hosmer & Lemeshow testing (HL test) was not significant indicating an acceptable fit between the predicted and actual outcomes.

### Detection of depression by doctors

**Figure 1:**
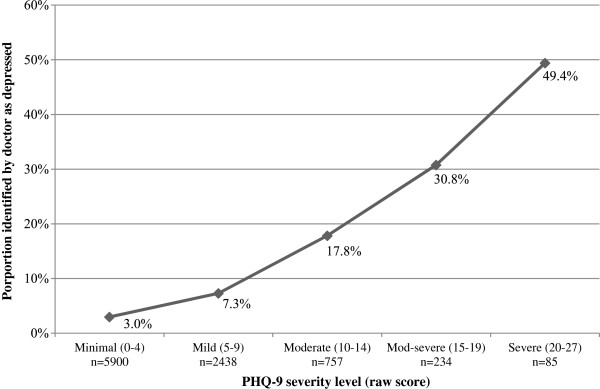
Detection of depression stratified by PHQ-9 sererity level.

The study doctors, blinded to their patients’ PHQ-9 screening scores, were asked to report whether they thought their patients had depression. Among all patients with PHQ-9 score >9 (*n* = 1076), 23.1% received a diagnosis of depression by the doctor. Detection rates for depression increased with higher PHQ-9 scores (Figure 1). Among patients with a moderately severe levels of depression (PHQ-9 score ≥ 15), 30.8% were diagnosed as having depression. Among patients with severe levels of depression (PHQ-9 score ≥ 20), 49.4% were diagnosed as having depression. In patients with a doctors diagnosis of depression and had a fully completed PHQ-9 score (*n* = 602), 58.6% screened negative for depression (PHQ-9 score ≤9).

### Predictors for diagnosis of depression by a doctor

Multiple logistic regression analysis was conducted to identify the predictors for doctor diagnosis of depression. As before, the significance of basic demographic variables and additional clinical characteristics were examined in different blocks. The distributions of each patient variable and multivariate statistics are shown in Table 4. Patient demographic factors significantly associated with higher likelihood of being diagnosed with depression included: being female, being aged 35 years and above; being unemployed, retired, or home-maker (relative to being employed); being non-Chinese, having no regular exercise pattern; having seen a doctor within the last 4 weeks; and having two or more co-morbidities. Patients most likely to receive a diagnosis of depression were unemployed (3.53-fold greater likelihood), or non-Chinese (2.52-fold greater likelihood).

**Table 4:** Associations between patient factors and doctor detection

**Patient factor**	**Detected, n (%)**	**Not detected, n (%)**	**χ2 (df)**	**Multiple logistic regression (*n*** **= 7346)**^**a**^	
				***Wald χ2 (df)***^**b**^**/Adj. OR (95% CI)**	
**Gender**			70.80 (1)**	23.70 (1)**	4.38 (1)*
Male	155 (25.5)	3795 (42.9)		1.00	1.00
Female	453 (74.5)	5053 (57.1)		1.89 (1.46 - 2.44)	1.35 (1.02 - 1.78)
**Age group**			34.76 (2)**	10.08 (2)**	7.94 (2)*
18-34 yrs	102 (17.1)	2351 (27.0)		1.00	1.00
35-54 yrs	216 (36.2)	3120 (35.9)		1.71 (1.23 - 2.39)	1.65 (1.14 - 2.39)
55+ yrs	279 (46.7)	3225 (37.1)		1.61 (1.05 - 2.49)	1.86 (1.14 - 3.01)
**Education**			23.68 (1)**	*0.17 (1)*	*0.03 (1)*
Secondary or above	407 (67.5)	6721 (76.3)		1.00	1.00
Primary or no formal education	196 (32.5)	2090 (23.7)		0.94 (0.71 - 1.25)	1.03 (0.75 - 1.41)
**Marital status**			1.73 (1)	*0.80 (1)*	*0.40 (1)*
Married	355 (59.1)	5422 (61.5)		1.00	1.00
Single/Separated/Divorced/Widowed	246 (40.9)	3391 (38.5)		1.11 (0.88 - 1.41)	0.92 (0.70 - 1.20)
**Working Status**			117.95 (4)**	38.85 (4)**	20.62 (4)**
Employed	260 (44.4)	5578 (64.0)		1.00	1.00
Unemployed	26 (4.4)	135 (1.5)		3.53 (2.06 - 6.05)	1.56 (0.78 - 3.13)
Retired	167 (28.5)	1827 (21.0)		2.00 (1.40 - 2.84)	2.16 (1.45 - 3.21)
House-maker	115 (19.6)	902 (10.3)		2.02 (1.46 - 2.80)	2.05 (1.41 - 2.97)
Student	18 (3.1)	275 (3.2)		1.62 (0.83 - 3.17)	1.18 (0.56 - 2.46)
**Household monthly income**			19.86 (1)**	*3.56 (1)*	*0.07 (1)*
More than HK$30,000	146 (28.5)	2947 (38.3)		1.00	1.00
HK30,000 or below	367 (71.5)	4746 (61.7)		1.27 (0.99 - 1.63)	1.04 (0.79 - 1.37)
**Ethnicity**			1.82 (1)	12.53 (1)**	
Chinese	603 (95.3)	8703 (96.3)		1.00	1.00
Non-Chinese	30 (4.7)	333 (3.7)		2.52 (1.51 - 4.20)	2.01 (1.11 - 3.63)
**Smoking habit**			1.16 (1)	*0.86 (1)*	*0.10 (1)*
Non- or ex-smoker	537 (88.5)	7656 (87.0)		1.00	1.00
Current smoker	70 (11.5)	1149 (13.0)		1.18 (0.83 - 1.67)	0.94 (0.64 - 1.39)
**Alcohol use**			4.99 (1)*	*0.13 (1)*	*0.00 (1)*
Less than once a week or never	548 (90.3)	7681 (87.2)		1.00	1.00
More than once a week	59 (9.7)	1131 (12.8)		1.07 (0.74 - 1.54)	1.00 (0.67 - 1.50)
**Regular exercise pattern**			20.92 (1)**	18.66 (1)**	10.58 (1)**
Yes	394 (65.1)	6465 (73.7)		1.00	1.00
No exercise at all	211 (34.9)	2313 (26.3)		1.62 (1.30 - 2.02)	1.51 (1.18 - 1.93)
**Seen a doctor in past 4 weeks**			65.72 (1)**	35.67 (1)**	
No	196 (31.0)	4302 (47.7)		1.00	1.00
More than once	436 (69.0)	4726 (52.3)		1.96 (1.57 - 2.44)	1.43 (1.12 - 1.83)
**Seen a TCM practitioner in past 4 weeks**			14.86 (1)**	*2.04 (1)*	*0.17 (1)*
No	469 (74.2)	7275 (80.5)		1.00	1.00
More than once	163 (25.8)	1758 (19.5)		1.20 (0.93 - 1.54)	0.94 (0.70 - 1.26)
**Disease co-morbidity**			65.21 (2)**	17.64 (2)**	*0.97 (2)*
None	221 (36.4)	4413 (50.1)		1.00	1.00
One	150 (24.7)	2216 (25.1)		1.29 (0.97 - 1.72)	1.05 (0.76 - 1.44)
Two or more	236 (38.9)	2188 (24.8)		1.88 (1.40 - 2.53)	1.18 (0.84 - 1.65)
**Family history mental illness**			70.56 (1)**	48.24 (1)**	9.53 (1)**
No	483 (81.5)	7919 (91.7)		1.00	1.00
Yes	110 (18.5)	721 (8.3)		2.61 (1.99 - 3.43)	2.61 (1.99 - 3.43)
**Service sector**			2042 (1)	*1.89 (1)*	4.34 (1)*
Public	183 (28.8)	2355 (26.0)		1.00	1.00
Private	453 (71.2)	6714 (74.0)		1.20 (0.93 - 1.55)	1.36 (1.02 - 1.83)
**Symptom severity/PHQ-9 score**^**c**^			779.72 (4)**		147.03 (4)**
Minimal/score 0-4	175 (29.1)	5725 (65.0)			1.00
Mild/score 5-9	178 (29.6)	2260 (25.6)			2.16 (1.63 - 2.86)
Moderate/score 10-14	135 (22.4)	622 (7.1)			4.67 (3.34 - 6.53)
Mod-severe/score 15-19	72 (12.0)	162 (1.8)			8.53 (5.41 - 13.46)
Severe/score 20-27	42 (7.0)	43 (0.5)			10.42 (5.20 - 20.89)
**Self-reported depression history**^**c**^			1564.17 (1)**		85.05 (1)**
No	314 (52.8)	8369 (95.7)			1.00
Yes	281 (42.6)	378 (4.3)			10.16 (7.73 - 13.34)
**Self-reported other mental illness history**^**c**^			330.67 (1)**		43.68 (1)**
No	450 (83.2)	8425 (97.6)			1.00
Yes	91 (16.8)	211 (2.4)			2.89 (1.93 - 4.31)

*p < .05; **p < .01.

^a^Model Chi-square = 872.43, df = 26, *p* < 0.001; Nagelkerke R-square = 0.320; Hosmer & Lemeshow Test, Chi-square = 17.79, df = 8, *p* = 0.023.

^b^Wald test statistics are shown in italic.

^c^Factors entered in second block of the regression model; Block Chi-sqaure = 591.50, df = 6, *p* < .001.

With all demographic variables retained in block 1, three additional patient variables were analyzed in the second block: PHQ-9 severity classification (minimal, mild, moderate, moderately severe and severe), patient-reported previous diagnosis of depression and patient-reported previous diagnosis of other mental health problem. Presence of moderately severe depression (PHQ-9 score of 20–27) was associated with an additional 8.53-fold increased likelihood of receiving a diagnosis of depression and the presence of severe depression (PHQ-9 score ≥20) was associated with a 10.42-fold increase. Having a patient-reported past diagnosis of depression was associated with a 10.16-fold increased likelihood of receiving diagnosis of depression, and having a patient-reported past history of other mental illness was associated with a 2.89-fold increase. Most patient demographic factors remained statistically significant after the inclusion of the block 2 variables with the exceptions of being unemployed and having ≥ two co-morbidities which changed from significant to insignificant, and service sector which changed from insignificant to significant. Multicollinearity among the factors analyzed was not evident. The whole set of predictors explained 32.0% of the model variance as suggested by Nagelkerke R-square value. A better model fit was supported by the significant model chi-square test, while the HL test became significant after the inclusion of second block variables.

### Management of depression

In patients with diagnosed depression, doctors were asked to record their management using a tick-box selection including medications, counselling, referral, observation and planned follow-up. Management was reported for 618 subjects. Overall, 50.6% of patients were prescribed medications (of which 84.0% were prescribed antidepressants) and 41.9%% provided counselling (of which 83% were provided with supportive therapy). Another health care provider was involved in 39.3% of patients: new referrals were provided in 8.6%, whilst 30.7% were already receiving care from another professional. The most common referral destinations were to a counselor (34.0%) or to a government-funded Hospital Authority specialist psychiatric service (22.6%). Only four patients (7.5% of those referred) were to a private psychiatrist.

Management patterns were slightly different in patients with and without a self- reported history of previous diagnosis of depression. Patients who reported a depression history were more likely having received care from another professional (46.0% vs. 17.8%), more likely be prescribed medication by the study doctor (59.1% vs. 41.6%), and less likely be observed (15.0% vs. 39.6%). Their PHQ-9 scores were significantly higher than those who reported no depression history (*t* = 2.19, *df* = 527, *p* = 0.029).

The PCPs management patterns are outlined in Tables 5, 6 and 7.

**Table 5:** Management of depression by doctors

**Depression management**	**n (%)**	**PHQ-9 mean score (SD)**
Patient currently under the care of another professional	189 (30.6%)	8.63 (6.49)
Being observed	172 (27.8%)	8.70 (5.85)
Follow-up scheduled	240 (38.8%)	9.22 (6.11)
Medication prescribed	313 (50.6%)	8.72 (6.33)
Counselling provided	259 (41.9%)	9.21 (6.27)
Referral to health care professional	53 (8.6%)	12.08 (6.45)

*Note*. Management data was not available for another18 patients who were detected with depression. Percentage totals may exceed 100% as more than one management could be provided.

**Table 6:** Breakdown of subcategories of management provided

**Breakdown of subcategory**	**n (%)**
**Medication prescribed (n=313)**	
Antidepressant	263 (84.0%)
Z-class drug	41 (13.1%)
Benzodiazepine	70 (22.4%)
Anti-psychotic	26 (8.3%)
**Counseling provided (n=259)**	
Supportive counseling	215 (83.0%)
Problem solving therapy	49 (18.9%)
Activity planning	31 (12.0%)
Cognitive behavioral therapy	36 (13.9%)
**Referral to health care professional (n=53)**	
Private psychiatrist	4 (7.5%)
Psychologist	7 (13.2%)
Government-funded psychiatric service	12 (22.6%)
Counsellor	18 (34.0%)
Emergency department	1 (1.9%)
Social worker	3 (5.7%)

*Note*. Percentage totals may exceed 100% due to multiple responses.

**Table 7:** Management of depression by doctors as classified by depression history

	**With depression history**	**No depression history**
	***n*** **=274**	***n*** **=303**
**Depression management (n, %)**		
Patient currently under the care of another professional	126 (46.0%)	54 (17.8%)
Being observed	41 (15.0%)	120 (39.6%)
Follow-up scheduled	106 (38.7%)	117 (38.6%)
Medication prescribed	162 (59.1%)	126 (41.6%)
Counselling provided	110 (40.1%)	129 (42.6%)
Referral to health care professional	23 (8.4%)	26 (8.6%)
Mean PHQ-9 score (Mean ± SD)	9.39 ± 6.35^†^	8.25 ± 5.87^†^

*Note*. Percentage totals may exceed 100% as more than one management could be provided.

^†^Independent t-test: *t* = 2.19, *df* = 527, *p* = 0.029.

## Discussion

This is the first territory-wide study examining the diagnosis and management of depression amongst Hong Kong’s primary care patients. A large scale survey such as this provides more accurate and actionable information when compared to surveys on selected clinical populations. Data on the rates of disease, barriers to care, identification of risk factors and service use is needed to help inform public policy and service planning [22]. This is one of the few studies performed in a predominantly Chinese population which includes both Chinese and non-Chinese subjects.

Although the prevalence of depressive disorders estimated in our study was similar to prevalence rates found in primary care settings in other parts of the world, by international standards, the detection rate of under one in every four patients who screened positive appears to be quite low [5]. Our study used a network of clinicians who had an active interest in mental health research. Of the 59 doctors who participated in the study, 14 had undertaken further diploma studies in community psychological medicine and all participants were aware that their patterns of diagnosis and management were being studied. Despite using this relatively biased sample of clinicians, detection rates were still lower than international standards suggesting that there may be factors aside from clinician skill which contribute to the low detection rates. It is well recognized that identification of depression in the primary care setting is challenging. Patients may not actively seek help or disclose any symptoms suggestive of a mood disorder, and the time available for the primary care consultation is often brief and mood disturbances may be missed [23]. PCPs often have difficulty in differentiating between psychological distress and psychiatric disorder due to difficulties in applying DSM-IV diagnostic criteria to primary care patients [24]. Relevant to our setting however is the further challenge of trying to identify depression in Chinese patients. The Chinese have been identified as an ethnic group with particularly low uptake rates of mental health services [25,26]. Chinese patients with depression often conceal their mood-related symptoms [27,28] and may either deny having any depressive symptoms or may express them more somatically [28]. Many elderly Chinese patients perceive having a low mood to be a normal part of aging, and would not consider reporting depressive symptoms to their clinician [29,30]. Studies conducted in Europe and in America have noted lower detection rates for patients of Chinese descent than in their non-Chinese counterparts [25,31]. This finding appears to be confirmed in our study. When controlling purely for demographic factors, non-Chinese patients had a 2.5-fold increased likelihood of being diagnosed with depression. Even when controlling for severity and patient- reported past history of depression and other mental illness, non-Chinese patients are still twice as likely to receive a diagnosis of depression by a doctor. In view of this finding, there may be a rationale to recommend screening of patients to help improve detection rates in Chinese patients.

Lack of continuity of care is another factor that can contribute to low detection rates. Studies have shown that many PCPs are reluctant to label a patient as depressed on a single consultation, but rather over a period of observation [32]. Many patients in Hong Kong do not have a regular doctor, opting for convenience and accessibility rather than continuity of care when seeking medical attention [13]. In a study on public sector primary care patients, only 10% reported to know their consulting doctor well [33]. In this study, patients who were most likely to receive a diagnosis of depression included those who were female, aged over 55 years, retired or housewives, and those who had seen a doctor within the last month. Patients with these characteristics are more likely to receive regular medical attention, making it easier for doctors to detect the presence of depressive symptoms [34].

In this study, it appears doctors in private practice may be more willing or able to diagnose depression. With symptom severity and patient-reported past history of depression controlled in the analysis, private sector patients were 1.36 times more likely to receive a depression diagnosis than those in the public sector. With an average consultation time of only 5.5 minutes [33], by international standards, primary care consultations in Hong Kong’s public sector clinics are brief [35]. As the main focus of these services is usually on chronic disease care, there may be insufficient time to explore psycho-social issues [13]. A recent meta-analysis found that consultations associated with a diagnosis of a psychological problem tended to be longer than those without any psychological diagnosis [36].

Our examination of doctors’ management patterns found over 50% of patients diagnosed with depression were prescribed medication. In Hong Kong, drugs are dispensed by the doctor rather than through a pharmacy which, in association with a lack of access to psychosocial services, are likely to be key reasons for the high rates of prescribing. Another reason for the high rates of drug prescribing may also be the doctor’s perception of patient expectations for medication. In a telephone survey of Hong Kong Chinese community members, although only 40% of respondents believed they always needed drugs to treat an illness, 76% expected to receive a prescription every time they saw a doctor. In almost 100% of cases, during their most recent doctor visit, at least one medication had been prescribed. The study concluded that doctors in Hong Kong over-estimate patients’ expectation for medications, and their prescribing habits may have subsequently produced a high expectation for medications by patients [37]. A more focused study is needed to better examine doctor’s prescribing practices and their perception of what constitutes best practice for managing primary care patients with depression.

Examination of the doctor’s referral patterns found that 8.6% of patients diagnosed with depression were referred for other services with 34% of new referrals to counselors, 23% to public hospital clinics and only 7.5% to private psychiatrists. As in many other settings, access to psychosocial services in Hong Kong is very limited. In addition, Hong Kong also has very few specialist psychiatrists. In 2005 the population to specialist ratio for psychiatrists in Hong Kong was 1:44,202, far higher than the UK where the ratio was 1: 16,836 [38]. Of these, only a small proportion of psychiatrists practice in the private sector [12]. In Hong Kong, patients can directly consult specialists in the private sector without a primary care referral. When asked who they would seek help from if they thought they were depressed, it has been reported that 20% of patients in Hong Kong would prefer to see a psychiatrist [39]. This has significant service implications as patients may potentially ‘by-pass’ seeing a primary care doctor, leading to excessive demands for already stretched specialist services.

### Strengths and limitations

One of the major strengths of this study was our success in recruiting a large number of primary care physicians to collaborate in this study. There are many service delivery options for patients seeking primary care in Hong Kong and our wide sampling of practice types captures this diversity. Despite this, there were limitations to our sampling strategy. As there is no comprehensive registry of primary care providers for Hong Kong, the mailing list of the Hong Kong College of Family Physicians (HKCFP) was chosen as the primary sample frame for the doctors. A limitation is that doctors who provide primary care, but are not members of the HKCFP were not deliberately sampled. The doctors who participated in this study were volunteers who joined our research network, and there was a biased sampling towards doctors with an interest in mental health. The results of this study are likely to reflect a “best case scenario” with better detection rates and more optimal treatment than is being offered in Hong Kong’s wider primary care setting. The low referral rates found in this study may have been a result of this sampling bias as a high proportion of the doctors had post-graduate training in psychological medicine and may have felt more capable of providing depression care than doctors without such training.

Whilst we recruited subjects from all three regions of Hong Kong, approximately 40% were recruited from Hong Kong Island, the smallest in terms of population size. Although the three regions differ in terms of geographic size, population number and socio-economy, a previous study of Hong Kong’s primary care found the morbidity patterns to be quite similar territory wide [40].

Although we were able to obtain a response rate of over 80%, no clinical information was available for non-respondents due to the lack of electronic medical records in most primary care clinics and ethical constraints on patient data privacy. Of the non-respondents 62.7% were female (vs 56.5% in respondents); 83.5% refused for no reason, 10.1% cited that they felt too sick, 6.1% cited that they did not have enough time, and 0.3% refused due to hearing difficulties.

Screening for depression was based on a subjective self-reported instrument and was not confirmed by a clinical diagnostic interview which would be the gold standard for diagnosis of depression.

## Conclusions

Around one in ten Hong Kong primary care patients screen positive for depression, in which doctors diagnose depression in approximately one in four. Patients with a past history of depression, who are unemployed, or who have multiple co-existing illnesses appear to be at greatest risk of being depressed. Patients with a past history of depression or who have severe symptoms of depression are more likely to receive a diagnosis of depression. Chinese patients are half as likely to receive a diagnosis of depression by a doctor as non-Chinese patients. In patients diagnosed with depression, over half are managed with medication. The most commonly used referral service by primary care doctors are to counselors. Further studies are needed to examine doctors prescribing practices and their perceptions of what constitutes best practice for managing primary care patients with depression.

## Competing interests

The authors declare that they have no competing interests.

## Authors’ contributions

CL initially conceived the study. All authors collectively designed and drafted the study protocol and sought funding and ethical approving. DF led on statistical analyses. CL, TPL, YL, BC, SW contributed to recruitment and data collection. KC was the project coordinator, recruited and trained the fieldworkers, assisted with recruitment of study doctors, coordinated the data collection, and contributed to the drafting of the manuscript. WYC is PI of the funding application, drafted the study protocol, coordinated the research network and research team, and drafted the manuscript. All authors have read the draft critically and approved the final manuscript.

## Pre-publication history

The pre-publication history for this paper can be accessed here:

http://www.biomedcentral.com/1471-2296/15/30/prepub
